# Supplementary material for: Acute Hypoxia Profile is a Stronger Prognostic Factor than Chronic Hypoxia in Advanced Stage Head and Neck Cancer Patients
Source: Cancers (Basel). 2019 Apr 25;11(4):583. doi: 10.3390/cancers11040583 (PMC6520712; doi:10.3390/cancers11040583)
Supplement: Supplementary file 1 [file cancers-11-00583-s001.zip › Supplementary Figures.pdf]

Supplementary Figure 1

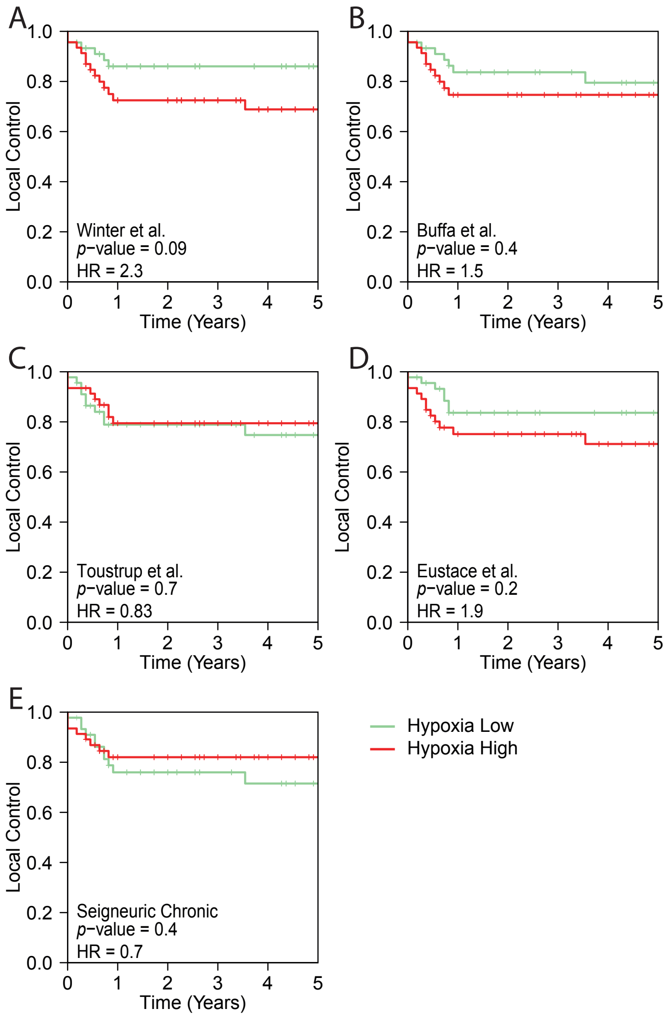

Figure S1. Univariable analyses of individual hypoxia profiles. A) Kaplan Meier curve of "High" versus "Low" hypoxia by Winter et al. in the Pramana cohort (N=91). The log-rank test was used to calculate the p-value. B, C, D, E depict the same as A except for Buffa, Toustrup, Eustace and Seigneuric Chronic, respectively.

Supplemental figure 2

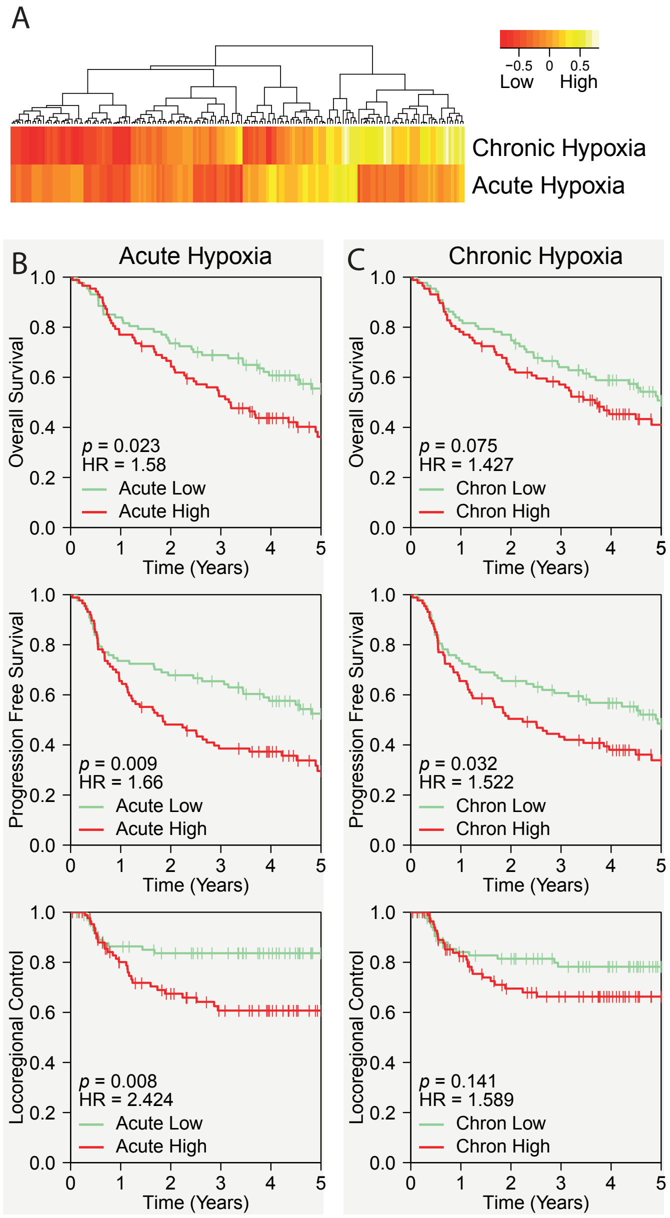

Figure S2. Distribution and outcome analyses of hypoxia in the Van der Heijden cohort. A) Heatmap of acute and chronic hypoxia scores. Seigneuric acute hypoxia represent acute hypoxia and chronic hypoxia is respresented by the joint chronic hypoxia score. B) Kaplan meier curves of Overall Survival, Progression Free Survival and Locoregional contol of acute hypoxa in the van der Heijden cohort (N=174). Statistics are calculated using a Cox proportional hazard model. A median split was used to divide patient into a "High" and a "Low" hypoxia group. C) Same as for B except for chronic hypoxia.

Supplementary figure 3

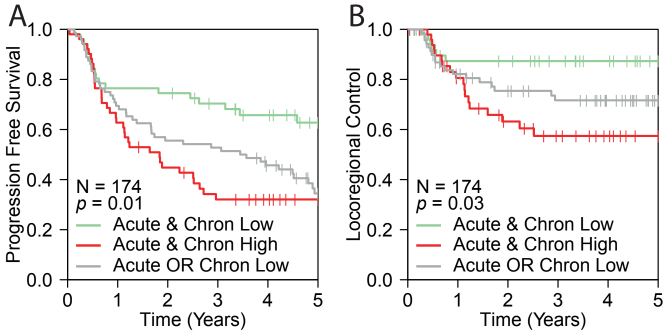

Figure S3. Combined acute and chronic hypoxia scores in the Van der Heijden cohort (N=174). A) Kaplan-Meier curve showing Progression Free Survival for 3 groups: (1) acute and chronic hypoxia both low, (2) acute or chronic hypoxia high, or (3) acute and chronic hypoxia both high. P-value represent the log-rank p-value. B) Same as A, except for locoregional control.
