# Supplementary material for: Acute Hypoxia Profile is a Stronger Prognostic Factor than Chronic Hypoxia in Advanced Stage Head and Neck Cancer Patients
Source: Cancers (Basel). 2019 Apr 25;11(4):583. doi: 10.3390/cancers11040583 (PMC6520712; doi:10.3390/cancers11040583)
Supplement: Supplementary file 1 [file cancers-11-00583-s001.zip › Supplementary Methods.docx]

**SUPPLEMENTARY METHODS DE JONG 2 *ET AL***

**Supplementary methods – mRNA extraction and sequencing of 34 larynx carcinomas.**

*mRNA extraction.*

RNA was extracted from pre-treatment biopsies using the Roche High Pure miRNA Isolation Kit (REF: 05080576001). In summary, 5 sections of 5 µm thick each were deparaffinized and macrodissected, to guarantee that the sample contained of at least 50% tumor cells. The RNA was further purified according to the manufacturer’s instructions.

*Cleanup/Total RNA quality control*

Quality and quantity of the total RNA was assessed with the 2100 Bioanalyzer using a Nano chip (Agilent, Santa Clara, CA). Following quantification, 1000 ng of each sample was subjected to an ethanol precipitation cleanup, ribosomal RNA depletion treatment and NGS library generation. Briefly, the samples were transferred to a 1.5-ml Eppendorf tube and the volume was adjusted to 180 μl, mixed with 18 μl of 3M Sodium Acetate with the addition of 1 μl of Glycogen (20 μg/μl) (Invitrogen, p/n 10814-010 ), followed by the addition of three volumes of ice-cold 100% ethanol , mixed and incubated overnight at -20 °C. The tubes were centrifuged at 13000 RCF for 1 hour and the supernatant discarded. The pellets were washed twice with 70% ethanol, air dried at room temperature for 10 minutes and dissolved in 30 μl of nuclease free water (Ambion p/n AM9937).

*Ribo-Zero treatment*

Ribosomal RNA sequences were depleted by treatment with the Ribo-Zero Magnetic Gold kit according to the manufacturer instructions (Epicentre, Cat no. MRZG12324), followed by an ethanol precipitation cleanup according to the manufacture instruction (Epicentre, Cat no. MRZG12324). The rRNA depleted fraction was subsequently used to generate NGS libraries suitable for the Illumina HiSeq2000 sequencing platform.

*TruSeq library preparation*

TruSeq cDNA libraries were generated using the TruSeq RNA Library Preparation Kit v2 sample preparation kit (Illumina Cat.No RS-122-2001/2) according to the manufacturer's instruction (Part # 15026495 Rev. D) with the following modifications. 1 μl of random primers (3 μg/μl), (Invitrogen p/n 48190-011) were added to the rRNA depleted fraction, followed by an incubation at 65 °C for 5 minutes in a thermocycler and placed on ice, after which the manufacturer’s protocol was further followed. Briefly, the RNA was reverse transcribed using SuperScript II Reverse Transcriptase (Invitrogen, part # 18064-014). Second strand synthesis was accomplished by using Polymerase I and RNaseH. The generated cDNA fragments were 3' end adenylated and ligated to Illumina Paired-end sequencing adapters and subsequently amplified by 15 cycles of PCR. The libraries were analyzed on a 2100 Bioanalyzer using a 7500 chip (Agilent, Santa Clara, CA), diluted and pooled equimolar into a 4-plex, 10 nM sequencing pool and stored at -20 °C.

*Sequencing*

The reads (51bp) were sequenced on a HiSeq2000 using V3 chemistry (Illumina Inc., San Diego), aligned against the human genome, build 37, using Tophat (version 2.0.6). Tophat allows to span exon-exon junctions. The samples were generated using a non-stranded library preparation protocol. Tophat was run with bowtie version 0.12.9 and supplied with a set of known gene models (GTF file, ensembl version 66) using the transcriptome index option. Other options supplied to Tophat were library typefr-unstranded, --prefilter-multihits and --no coverage. HTseq count was used to count the number of reads per gene. Only uniquely mapped reads were counted. Read counts of all samples were normalized to 10 million reads per sample. After normalization the expression values are log2 transformed. Upon log2 transformation, 1 was added to each expression value in order to avoid negative gene expression values.
