# Supplementary material for: Acute Hypoxia Profile is a Stronger Prognostic Factor than Chronic Hypoxia in Advanced Stage Head and Neck Cancer Patients
Source: Cancers (Basel). 2019 Apr 25;11(4):583. doi: 10.3390/cancers11040583 (PMC6520712; doi:10.3390/cancers11040583)
Supplement: Supplementary file 1 [file cancers-11-00583-s001.zip › Supplementary Tables.pdf]

Supplementary Table S1. Overview of the four clinical hypoxia gene expression profiles associated with patient outcome in HNSCC.

Year	Authors	Genes	Method used to identify the relevant genes
**2007**	Winter *et al* [25]	99	Clustering of genes around 10 well-known hypoxia associated genes
**2010**	Buffa *et al* [26]	51	Genes with similar expression pattern as well-known hypoxia associated genes in 3 HNSCC and 5 breast cancer series
**2011**	Toustrup *et al* [13]	15	In vitro experiments and correlation with Eppendorf hypoxia measurements
**2013**	Eustace *et al* [27]	26	Reduced variant of Buffa et al., validated in ARCON series

Supplementary Table S2. Genes in the used gene expression profiles

Winter	Buffa	Toustrup	Eustace	Seigneuric Acute	Seigneuric Chronic
ADORA2B	ACOT7	ADM	ALDOA	ACACA	ADAMTS13
AFARP1	ADM	ALDOA	ANGPTL4	ACOX1	ANGPTL4
AK3	AK3L1	ANKRD37	ANLN	ACSS1	ARID5B
ALDOA	ALDOA	BNIP3	BNC1	AIF1	ARL6IP1
ANGPTL4	ANKRD37	BNIP3L	C20orf20	AMH	C8orf4
ANKRD9	ANLN	C3orf28	CA9	ATF3	CASC2
ANLN	BNIP3	EGLN3	CDKN3	ATP5S	CDKN2D
AWR	C20orf20	KCTD11	COL4A6	ATXN7L1	CEP250
B4GALT2	CA9	LOX	DCBLD1	BACH1	CNOT2
BCAR1	CDKN3	NDRG1	ENO1	BET1L	CRABP2
BMS1L	CHCHD2	P4HA1	FAM83B	BTBD7	CSRP2
BNIP3	CORO1C	P4HA2	FOSL1	C12orf30	DDIT4
C14orf156	CTSL2	PDK1	GNAI1	C12orf53	DGCR8
C15orf25	DDIT4	PFKFB3	HIG2	CCL26	DOCK1
C20orf20	ENO1	SLC2A1	KCTD11	CCNH	DPYSL2
CA12	ESRP1		KRT17	CCT2	EHHADH
CA9	GAPDH		LDHA	CENTB2	EIF4E3
CDCA4P	GPI		MPRS17	COL6A3	ENO1
COL4A5	HIG2		P4HA1	CPEB4	ENO2
CORO1C	HK2		PGAM1	DKFZP761H1	FAM83A
CTEN	KIF20A		PGK1	DUSP3	FANCC
DKFZP564D166	KIF4A		SDC1	EIF4EBP2	FNDC3B
DPM2	LDHA		SLC16A1	FAM92A1	GPR75
EIF2S1	LRRC42		SLC2A1	GAS6	HIST1H4C
GAPD	MAD2L2		TPI1	GATAD2B	HNRPAO
GMFB	MAP7D1		VEGFA	GFOD2	INSIG1
GSS	MCTS1			GRK6	JUN
HES2	MIF			GRM3	KALRN
HIG2	MRPL13			HIST1H2AL	KEAP1
HOMER1	MRPL15			HMMR	KIF1B
HSPC163	MRPS17			IFI6	KIRREL
IL8	NDRG1			IGF1R	KRT8
IMP-2	NP			IGFBP1	LOC645619
KCTD11	P4HA1			IGSF11	LOX
KIAA1393	PFKP			JMJD2A	MKNK2
KRT17	PGAM1			KIAA1219	MLL3
LDHA	PGK1			LOC64645 0	MXI1
LDLR	PSMA7			LOC728488	MYADM
LOC149464	PSRC1			LY86	NAV2
LOC56901	SEC61G			MAML2	NDRG1
LRP2BP	SHCBP1			NCAPH2	PCTK3
MGC14560	SLC16A1			NIT1	PDK1
MGC17624	SLC25A32			NSD1	PFKP
MGC2408	SLC2A1			OSTM1	PGK1
MGC2654	TPI1			PAPPA	PIM3
MIF	TUBA1B			PARG	PLOD1
MNAT1	TUBA1C			PCF11	PPP1R3B
MRPL14	TUBB6			PCSK1	PRKCB1

MRPS17	UTP11L			PEX14	PRKCBP1
MTX1	VEGFA			PHF10	PTGES
NDRG1	YKT6			PIK3R4	RAVER2
NME1				PRKAG2	SERINC5
NTMT1				PTPLAD1	SLC6A10P
NUDT15				RAB4B	SLC6A8
P4HA1				RBM4	SLCO4A1
PDZK11				RBPMS	SMEK1
PFKFB4				RHOBTB3	STRBP
PGAM1				RNASE4	SYTL2
PGF				SLC5A12	TCBA1
PGK1				SOX12	NEAT1
PLAU				SSH2	TSC22D2
PLEKHG3				ST3GAL1	UHRF1
PPARD				TERT	WSB1
PPP2CZ				TIMP2	ZFHX1B
PPP4R1				TLE3	ZNF511
PSMA7				TP53111	
PSMB7				TRNT1	
PSMD2				TTLL5	
PTGFRN				ZNF117	
PVR				ZNF664	
PYGL					
RAN					
RNF24					
RNPS1					
RUVBL2					
S100A10					
S100A3					
SIP1					
SLC16A1					
SLC2A1					
SLC6A10					
SLC6A8					
SLCO1B3					
SMILE					
SNX24					
SPTB					
TEAD4					
TFAP2C					
TIMM23					
TMEM189					
TMEM30B					
TPBG					
TPD52L2					
TPI1					
TUBB2					
VAPB					
VEGF					
VEZT					
XPO5					

Supplementary Table S3. Patient characteristics Pramana cohort: 91 HNSCC stage III-IV chemoradiotherapy patients.

Variable	No of patients	%
**Age (years)**		
Mean	58	
Range	29-79	
**Sex**		
Male	61	67
Female	30	33
**Tumor site**		
Hypopharynx	20	22
Larynx	7	8
Oral cavity	13	14
Oropharynx	51	56
**T-stage**		
1	1	1
2	5	5
3	32	35
4	53	58
**N-stage**		
0	24	26
1	10	11
2	50	55
3	7	8
**Local recurrences**		
No	72	79
Yes	19	21
**Median time (years)**		
To local recurrence	0.5	
Follow-up without	4.3	

Supplementary Table S4. Patient characteristics de Jong 1 cohort: 99 larynx/oropharynx radiotherapy patients.

Variable	No of patients	%
**Age (years)**		
Mean	62.1	
Range	42.3-85.5	
**Sex**		
Male	84	85
Female	15	15
**Tumor site**		
Larynx	86	87
Oropharynx	13	13
**T-stage**		
1	22	22
2	40	40
3	35	35
4	2	2
**N-stage**		
0	83	84
1	8	8
2	6	6
3	2	2

Supplementary Table S5. Patient characteristics de Jong 2 cohort:
34 larynx radiotherapy patients.

Variable	No of patients	%
**Age (years)**		
Mean	67.2	
Range	40.0-85.0	
**Sex**		
Male	20	59
Female	14	41
**Tumor site**		
Larynx	34	100
**T-stage**		
2	22	65
3	12	35
**N-stage**		
0	26	76
1	1	3
2	7	21

Supplementary Table S6. Patient characteristics of the van der Heijden cohort (NKI-CRAD and DESIGN combined): 174 advanced stage HPV negative HNSCC patients treated with chemoradiotherapy. Age at diagnosis and follow-up time in years.

Variable		No of patients	%
**Age (years)**			
	Median	61	
	Range	40-80,2	
**Sex**			
	Male	127	73
	Female	47	27
**Tumor site**			
	Larynx	31	18
	Hypopharynx	66	38
	Oropharynx	77	44
**Stage**			
	I-III	35	20
	IVA-IVB	139	80
**T-stage**			
	T1-3	110	63
	T4	64	37
**N-stage**			
	N0-N2a	62	3
	N2b-N3	112	64
**Tumor Volume in cc. (N=149)**	Median	23,6	
	Range	2,3-750	
**Total cisplatin dose < 200**	Yes	63	37
	No	107	63
**Local Recurrences**			
	No	146	84
	Yes	28	16
**Follow up time (median years)**	To local recurrence	0.71	
	Without local recurrence	4.00	

Supplementary Table S7. Univariate Cox proportional hazard analysis of parameters with patient outcome in the Van der Heijden cohort. OS: Overall Survival; PFS: Progression Free Survival; LC: Local Control; LRC: Locoregional Control; DM: Distant Metastasis Free Survival; HR: Hazard Ratio.

	OS		PFS		LC		LRC		DM	
**Variable**	**HR**	***p*-value**	**HR**	***p*-value**	**HR**	***p*-value**	**HR**	***p*-value**	**HR**	***p*-value**
**Gender**										
Female	0,7	0,12	0,72	0,14	0,41	0,1	0,4	**0,037**	0,3	**0,025**
Male	REF		REF		REF		REF		REF	
**Age at diagnosis**	1,01	0,575	1	0,75	1,01	0,836	0,99	0,6	0,98	0,46
**Tumor site**										
Oropharynx	REF		REF		REF		REF		REF	
Hypopharynx	0,54	**0,005**	0,56	**0,007**	0,77	0,56	0,83	0,61	0,44	**0,04**
Larynx	0,57	0,07	0,8	0,43	1,6	0,31	1,7	0,18	0,94	0,87
**Disease stage**										
II-III	0,56	**0,033**	0,51	**0,014**	0,7	0,48	0,54	0,17	0,29	**0,04**
IVA-IVB	REF		REF		REF		REF		REF	
**T classification**										
T1-T3	REF		REF		REF		REF		REF	
T4	1,18	0,4	1,25	0,26	1,22	0,609	0,82	0,55	1,03	0,94
**N classification**										
N0-N2a	0,81	0,3	0,86	0,44	0,91	0,81	1,03	0,92	0,46	**0,049**
N2b-N3	REF		REF		REF		REF		REF	
**Cumulative cisplatin < 200**										
No	REF		REF		REF		REF		REF	
Yes	1,84	**0,003**	1,77	**0,004**	5,2	**0,0001**	2,74	**0,002**	1,2	0,58
**Tumor Volume**	1,006	**5,81E-07**	1,006	**8,03E-06**	1,006	0,26	1,005	0,26	1,009	**0,022**
**Acute Hypoxia**	2,36	**0,029**	2,66	**0,009**	6,07	**0,008**	5,51	**0,003**	3,7	**0,035**
**Chronic Hypoxia**	1,37	0,17	1,48	0,07	1,96	0,121	2,21	**0,027**	1,31	0,47

Supplementary Table S8. 10 hypoxia 'seed genes' and correlation with in vitro Seigneuric acute and Seigneuric chronic hypoxia profiles

Correlation with *in vitro* :	10 seed genes										Average correlation
	ADM	SLC2A1	PDK1	ENO1	HK2	PFKFB3	AK3	CCNG2	CA9	VEGF	
**Seigneuric Acute**	-0.22	-0.17	-0.05	0.03	-0.18	-0.15	0.05	0.13	-0.05	-0.04	-0.06
**Seigneuric Chronic**	0.48	0.45	0.23	0.48	0.36	0.17	-0.27	0.06	0.24	0.32	0.25
